# Adult dietary patterns with increased bean consumption are associated with greater overall shortfall nutrient intakes, lower added sugar, improved weight-related outcomes and better diet quality

**DOI:** 10.1186/s12937-024-00937-1

**Published:** 2024-03-20

**Authors:** Yanni Papanikolaou, Joanne Slavin, Victor L. Fulgoni

**Affiliations:** 1 Nutritional Strategies, Nutrition Research & Regulatory Affairs, 59 Marriott Place, Paris, ON N3L 0A3 Canada; 2 Department of Food Science and Nutrition, College of Food, Agricultural, and Natural Resource Sciences, University of Minnesota, 1334 Eckles Ave., St. Paul, MN 55108 USA; 3 Nutrition Impact, Nutrition Research, 9725 D Drive North, Battle Creek, MI 49014 USA

**Keywords:** Beans, Public health, Shortfall nutrients, Diet quality, NHANES

## Abstract

**Background:**

Limited evidence is available that focuses on beans within American dietary patterns and health. The purpose of this study was to identify commonly consumed adult dietary patterns that included beans and compare shortfall nutrient intakes and diet quality, relative to adults whose typical dietary pattern did not include beans.

**Methods:**

The analyses used data from the National Health and Nutrition Examination Survey, 2001–2018. Cluster analysis was used to identify bean patterns of consumption, while the USDA food coding system defined daily beans consumed. Five bean dietary patterns of consumption were identified, of which four patterns included both canned beans and dry beans, while one pattern had no bean consumption. Bean consumption was defined as those consuming kidney beans, black beans, chickpeas, and/or pinto beans.

**Results:**

Adults consuming Bean Dietary Patterns 1, 2, 3 and 4 had significantly higher diet quality scores (as assessed by USDA’s Healthy Eating Index-2015) compared to the no-bean pattern (61.2 ± 0.5, 58.9 ± 0.5, 55.2 ± 0.4 and 56.5 ± 0.8 vs 48.8 ± 0.2 *p*’s < 0.0001). Bean consumers also had significantly higher intakes of several shortfall nutrients (choline, alpha-linolenic acid, folate, iron, magnesium and vitamin E) relative to non-consumers of beans. Similarly, intake of dietary fiber, potassium and calcium, all nutrients of public health concern were significantly higher in bean patterns compared to no-beans. Bean Dietary Pattern 1 (~ 13.5% of total daily kcal from beans or ~ 2 servings of beans/day) and 2 (~ 9.5% of total daily kcal from beans or ~ 1.7 servings of beans/day) were significantly associated with lower BMI, decreased body weight and improved waist circumference relative to no-beans.

**Conclusions:**

Dietary patterns that are rich in canned and dry beans were associated with significantly higher diet quality scores and greater intake of shortfall nutrients, including nutrients of public health concern. Bean dietary patterns were also associated with improved weight-related outcomes. Dietary guidance should consider the nutrient and health benefits associated with the promotion of increased canned and dry bean consumption in American dietary patterns.

## Background

Dietary patterns that contribute optimal nutrition while concurrently supporting the environment and mitigating climate change is becoming increasingly relevant globally. Beans, and other protein-rich legumes are considered one of the lowest climate impacting foods via reduced greenhouse gas emissions [1], removal of carbon from the atmosphere during production and improved soil fertility through nitrogen fixation [2, 3]. Additionally, the inclusion of beans within American dietary patterns has routinely been recommended by current [4] and previous Dietary Guidelines for Americans (DGA) [5]. Indeed, beans contribute nutrient density (i.e., provide a meaningful daily contribution as recommended by National Academies Sciences, Engineering and Medicine, Institute of Medicine’s Dietary Reference Intakes; https://nap.nationalacademies.org/catalog/11537/dietary-reference-intakes-the-essential-guide-to-nutrient-requirements) to dietary patterns, with a 100 g serving (115–164 kcal) of cooked beans contributing 5.3–10 g of dietary fiber, 111–168 mg of potassium, 83–207 mcg of folic acid, 17–70 mg of calcium, 7.7–9.3 g of protein, 0.4–2.6 g of total fat, and 21–27 g of carbohydrate [6]. A recent panel of experts examining the evidence surround carbohydrate food quality reported fruits, vegetables, beans and other legume products scored the highest quality carbohydrate score predominantly due to the contribution of higher dietary fiber and potassium levels and lower amounts of sugar and sodium levels [7–9]. Beans, peas, and lentils are unique foods in that they can be considered a part of the protein foods group as well as the vegetable group. DGA has previously stated “shifts are needed within the protein foods group to add variety and selecting from the seafood subgroup or the beans, peas, and lentils subgroup more often could help meet recommendations while still ensuring adequate protein consumption” [4]. Nonetheless, less than 20% of the population are at or above recommendations for bean, peas and lentil consumption [4]. Beans, peas, and legumes are consumed in relatively small amounts, at an average of 0.1 cup eq/day [10]. Canned beans account for approximately 75% of units sold at US retail, outselling other forms by roughly 4:1 [11].

Previous work using NHANES 1999–2002 has shown numerous nutrient and health outcome benefits associated with both canned and dry bean consumption. In particular, adults consuming baked beans had reduced systolic blood pressure versus non-consumers, even in the presence of higher daily sodium intakes. Similarly, adults reporting consumption of a variety of beans (pinto, kidney, etc.) had greater intakes of shortfall nutrients, and improved weight-related outcomes, relative to bean non-consumers. The inclusion of both baked beans and a variety of beans in dietary patterns was associated with reduced intake of discretionary fat and added sugars [12]. A recent systematic review and meta-analysis of randomized clinical trials further substantiates the health benefits stemming from bean consumption, such that beans significantly reduced LDL-cholesterol, cardiovascular disease (CVD) and coronary heart disease (CHD) risk [13]. Similarly, data from a large cohort showed legume consumption four times or greater per week, was associated with significantly reduced risk for CVD and CHD in adults, leading the investigators to argue in support of increasing legume consumption as a key dietary strategy to help prevent CHD in the general population [14]. Others have shown that bean consumption does not impact cardiovascular risk factors, however, the intervention period has also been questioned as being short in duration [15].

The nutrient-density of beans [16, 17] and their contributions to diet quality have been documented by the most recent collaboration between the National Cancer Institute (NCI) and the United States Department of Agriculture (USDA) to update the Healthy Eating Index (HEI) diet quality scale. The sub-component of ‘greens and beans’ allow for a maximum score from this component when an individual has ≥ 0.2 cup equivalents per 1000 kcal and a score of zero if the dietary patten contains no dark green vegetables or legumes (i.e., beans and peas) [18]. While the average diet quality has slightly improved in the last decade, scores indicate that diet quality is not aligned with DGA recommendations [4, 5], yet accumulating evidence exists supporting increased fruit, legumes and vegetables and improvements in diet quality and longevity [19–23].

As current dietary guidance includes beans, peas, and lentils as a component of the core elements that comprises a healthy dietary pattern and includes these foods within both the vegetable and protein food groups, the purpose of the current analysis was to identify how beans are consumed within American dietary patterns and determine relationships with nutrient intakes and diet quality in adults.

## Methods

The analysis used data from What We Eat in America (WWEIA), which represents the dietary intake component of NHANES. NHANES is a cross-sectional, nationally representative survey directed by the National Center for Health Statistics. NHANES samples free-living, non-institutionalized individuals and is currently a continuous study complied by the Centers for Disease Control and Prevention (CDC) where data are released every two years [24, 25]. Ethical protocols, including informed consent from study participants have been previously obtained, approved and documented by the CDC ethic boards. The distribution of the US population, in addition to response rates and population totals for NHANES are summarized by the CDC [26]. Data for the nutrients examined are from the U.S. Department of Agriculture (USDA) Food and Nutrient Database for Dietary Studies (FNDDS) database for NHANES [27]. The FNDDS databases determine food and beverage nutrient values in WWEIA. The collection procedure for WWEIA involves use of the Automated Multiple Pass Method (AMPM), representing a dietary collection tool that provides a valid, evidence-based approach for gathering data for national dietary surveys [28, 29]. Although two days of recall are recorded in NHANES, the current analysis focused on 24-h recalls obtained from Day 1 which were collected via an in-person interview. Accuracy, effectiveness, and efficiency of the AMPM method has been extensively reported and previously documented [29].

Bean patterns of consumption were determined using SAS 9.4 (SAS Institute, Cary, NC, USA) PROC CLUSTER using the first day of 24-h dietary recall from NHANES 2001–2018 and appropriate population weights. The cluster analysis approach is a statistical procedure that analyses large datasets to identify various dietary patterns while maximizing differences among the dietary patterns. Cluster analysis allows for the focus on a specifically defined aspect (i.e., bean consumption) and then directs maximal differences in clusters for evaluations. For these analyses bean consumption was defined to include kidney beans (i.e., white and red), black beans, chickpeas, and pinto beans (canned and non-canned), while soybean consumption was excluded in the analysis. For the present analyses, cluster analysis allowed for group comparisons rather than factor analysis which examines associations.

USDA classifies foods into 15 main food groupings, about 45 food subgroups and over 150 separate food categories [30]. For the present analyses, food groups were collapsed into the 14 WWEIA food groups (excluding the baby food group) and beans as defined by the bean definition. All food codes fit in one and only one of the food pattern groupings. The patterns identified by the cluster analysis were then identified by percent calories within each food pattern grouping (only foods that contributed 3% or more of calories were included) at the centroid of each cluster. Using this method resulted in 4 readily identifiable bean patterns (refer to Table 1). In addition, a ‘no consumption’ of beans group (i.e., no beans reportedly consumed throughout the 24-h reporting session) was identified, thus creating a total of 5 unique patterns of consumption for evaluation. With food patterns identified, each participant was placed into one bean pattern of consumption. The cluster definitions and the associations of subjects with a cluster were directly from the output of the cluster procedure and each subject was placed in the cluster that matched most closely to the pattern of calories across the food categories.

**Table 1:** Percent energy (kcal) from bean patterns (Clusters) of consumption in US adults, ≥ 19 years-old

Food Group	No Bean Dietary Pattern (*N* = 38,159)	Bean Dietary Pattern 1 (*N* = 2,861)	Bean Dietary Pattern 2 (*N* = 1,216)	Bean Dietary Pattern 3 (*N* = 1,768)	Bean Dietary Pattern 4 (*N* = 542)
Beans	0	13.5 (~ 2 servings of beans)	9.5 (~ 1.7 servings of beans)	9.3 (~ 1.7 servings of beans)	9.6 (~ 1.7 servings of beans)
Dairy Milk	6.6	7.5	3.9	5.1	5.7
Protein Foods	15.3	14.4	27.9	6.7	9.0
Mixed Dishes	21.2	6.2	8.1	36.7	7.4
Grains	13.2	23.2	9.6	9.3	9.7
Sweets/Snacks	15	8.4	12.5	12.4	36.6
Fruit	2.8	3.6	1.9	2.6	2.1
Vegetables	5.7	5.3	5.8	3.1	3.5
Beverages	9.7	10.6	6.4	7.2	9.6
Alcohol	4.2	1.5	9.3	3.3	1.8

All values represent % calories contributed to the total dietary pattern; NHANES 2001–2018

Means (± standard errors) for daily nutrient intakes, food group consumption, and diet quality were determined using day 1 dietary intake data. Diet quality was assessed using USDA’s 2015 Healthy Eating Index (HEI) [18]. HEI is a validated dietary tool that provides a measure of diet quality and conformance to US dietary guidance [18].

Covariates for analyses of nutrient/energy intake, HEI-2015 and HEI-2015 sub-components were age, gender, ethnicity, and household poverty income ratio (PIR). PIR was grouped into three categories (< 1.25, 1.25–3.49, and > 3.49) and was based the US federally established poverty criteria, thus a PIR of < 1.25 equated to below 125% of poverty level, while higher values represented the subject was from a household with higher income status. Nutrient and food group intakes were adjusted for covariates mentioned above, but also for energy intakes. The main comparison of interest was to compare results between the various bean patterns of consumption identified and the no bean consumption dietary pattern group. A *p*-value of ≤ 0.05 was used to determine statistical significance in the current analysis.

## Results

### Bean patterns of consumption in adults

Four bean foods patterns of consumption (i.e., clusters) were identified in the present analysis, one of which included adults who did not consume any beans during the first dietary recall. Table 1 lists bean patterns isolated in adults, with the highest bean consumption seen in Bean Dietary Pattern 1 (13.5% of daily calories stemming from beans) and the lowest bean consumption seen in Dietary Pattern 3 (9.3% of daily calories originating from beans). Bean Dietary Pattern 4 had the greatest contribution of calories (36.6% of daily calories) stemming from sweets/snacks (i.e., cakes, cookies, pies, doughnuts, potato chips, candy, etc.), while Bean Dietary Pattern 3 had 36.7% of all calories derived from mixed dishes—a category which includes mixed meals (i.e., Mexican burrito and taco dishes, Asian fried rice, pizza, hamburgers, hot dogs, cold cut sandwiches, macaroni and cheese, etc.), which have been known to contribute greater amounts of sodium and saturated fat. Bean Dietary Pattern 1 and 2 had the largest caloric contributions from vegetables, (5.8% of total daily calories).

### Nutrient and energy intake within bean patterns of consumption

Mean energy and nutrient intakes are summarized in Table 2. Daily energy intake was higher in 3 of the 4 bean patterns relative to the no beans pattern, however, numerous statistically significant and favorable outcomes were observed both in macro- and micronutrient intakes after adjusting for energy intake. While overall carbohydrate intake was elevated in 3 of the 4 bean patterns of consumption, added and total sugars were significantly lower in Bean Dietary Pattern 2 and 3 compared to the no bean pattern. Added and total sugars were signficantly greater in Bean Dietary Pattern 4, likely due to the greater consumption of sweets and snacks in this group (see Table 1). Daily protein intake was significantly greater in two of the bean clusters and daily total and saturated fat intake was higher in three of the bean clusters when compared to the no bean group. Adults in Bean Dietary Pattern 1, which represented the bean cluster with the greatest calories sourced from beans, had significantly lower total fat and saturated fat intake relative to the no bean cluster. Total monounsaturated and polyunsaturated fatty acids were significantly elevated in 3 of the 4 bean patterns of consumption compared to no beans.

**Table 2:** Adjusted mean (SE) nutrient and energy intake for all bean and no-bean clusters

**Energy/Macronutrients**	**No Beans**		**Bean Dietary Pattern = 1**			**Bean Dietary Pattern = 2**			**Bean Dietary Pattern = 3**			**Bean Dietary Pattern = 4**		
	**Cluster 0**		**Cluster 1**			**Cluster 2**			**Cluster 3**			**Cluster 4**		
	**LSM**	**SE**	**LSM**	**SE**	**P**	**LSM**	**SE**	**P**	**LSM**	**SE**	**P**	**LSM**	**SE**	**P**
Energy (kcal)	2066	10.0	2087	27.0	0.464	2360	48.0	< 0.0001	2372	32.0	< 0.0001	2502	57.0	< 0.0001
Carbohydrate (g)	252	1.0	287	4.0	< 0.0001	247	5.0	0.351	294	4.0	< 0.0001	348	8.0	< 0.0001
Total sugars (g)	114	1.0	118	2.0	0.117	97.2	3.0	< 0.0001	108	2.0	0.023	171	5.0	< 0.0001
Added Sugar (tsp eq)	17.9	0.2	16.6	0.6	0.023	15.2	0.6	< 0.0001	15.9	0.5	< 0.0001	29.5	1.2	< 0.0001
Protein (g)	79.8	0.4	82.9	1.3	0.023	98.6	2.2	< 0.0001	92.2	1.4	< 0.0001	80.5	2.2	0.725
Total fat (g)	78.1	0.5	69.2	1.2	< 0.0001	93.3	2.5	< 0.0001	89.1	1.6	< 0.0001	89.4	2.8	0.0001
Total monounsaturated fatty acids (g)	28.1	0.2	25.3	0.5	< 0.0001	35.5	1.0	< 0.0001	32.3	0.6	< 0.0001	32.4	0.9	< 0.0001
Total saturated fatty acids (g)	25.2	0.2	21.0	0.4	< 0.0001	27.3	0.8	0.010	28.7	0.6	< 0.0001	28.0	1.0	0.010
Total polyunsaturated fatty acids (g)	17.6	0.1	16.7	0.3	0.0040	22.4	0.7	< 0.0001	20.2	0.4	< 0.0001	21.2	0.9	0.0001
Cholesterol (mg)	297	3.0	262	7.0	< 0.0001	395	13.0	< 0.0001	293	9.0	0.607	256	12.0	0.0006
Dietary fiber (g)	14.9	0.1	26.3	0.5	< 0.0001	22.0	0.5	< 0.0001	26.2	0.4	< 0.0001	24.7	0.8	< 0.0001
**Vitamins/Minerals**														
Calcium (mg)	866	6.0	906	17.0	0.025	774	23.0	0.0002	1031	21.0	< 0.0001	945	43.0	0.0620
Magnesium (mg)	276	1.0	352	5.0	< 0.0001	361	7.0	< 0.0001	350	5.0	< 0.0001	354	12.0	< 0.0001
Phosphorus (mg)	1285	7.0	1421	21.0	< 0.0001	1504	31.0	< 0.0001	1524	23.0	< 0.0001	1417	45.0	0.003
Iron (mg)	14.1	0.1	17.1	0.3	< 0.0001	15.7	0.4	< 0.0001	17.7	0.3	< 0.0001	17.2	0.6	< 0.0001
Zinc (mg)	10.9	0.1	12.1	0.2	< 0.0001	13.5	0.4	< 0.0001	12.9	0.2	< 0.0001	12.5	0.5	0.002
Sodium (mg)	3422	17.0	3476	53.0	0.336	3834	89.0	< 0.0001	4254	70.0	< 0.0001	3731	107.0	0.004
Potassium (mg)	2491	11.0	3047	42.0	< 0.0001	3052	54.0	< 0.0001	3056	45.0	< 0.0001	2991	68.0	< 0.0001
Folate, DFE (mcg)	500	3.0	639	12.0	< 0.0001	511	14.0	0.4040	625	12.0	< 0.0001	571	24.0	0.0030
Riboflavin (Vitamin B2) (mg)	2.0	0.01	2.0	0.04	0.289	1.9	0.04	0.174	2.0	0.04	0.140	2.1	0.1	0.229
Niacin (mg)	24.8	0.2	22.8	0.5	< 0.0001	26.8	0.7	0.003	24.6	0.5	0.638	22.3	1.0	0.011
Thiamin (Vitamin B1) (mg)	1.5	0.01	1.7	0.03	< 0.0001	1.6	0.04	0.045	1.8	0.03	< 0.0001	1.7	0.2	0.171
Total choline (mg)	326	2.0	345	7.0	0.009	460	12.0	< 0.0001	358	8.0	0.0001	333	12.0	0.534
Vitamin A, RAE (mcg)	580	9.0	595	38.0	0.630	511	27.0	0.0080	580	19.0	0.999	617	36.0	0.289
Vitamin B12 (mcg)	5.0	0.1	4.8	0.4	0.476	4.7	0.2	0.045	4.6	0.2	0.017	4.1	0.3	0.003
Vitamin B6 (mg)	2.0	0.02	2.1	1.0	0.001	2.2	1.0	0.000	2.1	1.0	0.048	2.0	1.0	0.340
Vitamin C (mg)	86.7	1.1	106.0	4.0	< 0.0001	82.2	3.1	0.141	94.9	2.9	0.006	94.3	6.2	0.222
Vitamin D (D2 + D3) (mcg)	4.3	1.0	4.9	0.3	0.018	4.7	0.3	0.219	3.8	0.1	0.0002	4.0	0.3	0.321
Vitamin E as alpha-tocopherol (mg)	7.6	1.0	7.6	0.2	0.8	9.2	0.4	< 0.0001	9.1	0.2	< 0.0001	9.8	0.5	< 0.0001

Covariates include age, gender, ethnicity, poverty income ratio, and for all variables except Energy, the covariate of energy (kcal)

NHANES 2001–2018, ≥ 19 Years-Old

*LSM* Least square mean, *SE* Standard error, *P p* value of difference as compared to cluster 0 (No Beans Dietary Pattern)

When considering dietary fiber and potassium, both identified as public health nutrients of concern by DGA, both nutrients were substantially higher in all four bean deiatry patterns compared the dietary pattern that excluded beans. Other shortfall nutrients, including magnesium, folate DFE, iron and vitamin E, were significantly higher in several dietary patterns that included beans when compared to the dietary patterns without daily bean intake. While no signifiant differences were observed between Bean Dietary Pattern 1 and the no bean pattern, the remaining bean patterns of consumption all showed elevated daily sodium relative to the no bean pattern. Total choline, while underconsumed in the US population, was significantly higher in adults consuming Bean Dietary Pattern 1, 2 and 3 in comparison to adults in the no bean pattern of consumption.

### Diet quality scores within bean patterns of consumption

Table 3 provides a measure of diet quality scores, as measured by HEI-2015, within each bean pattern of consumption identified and compares all bean patterns to the no bean pattern of consumption. All bean dietary patterns idenified showed significantly higher total diet quality scores compared to the no bean dietary pattern of consumption. Moreover, adults in all four bean dietary patterns consumed significantly greater amounts of total vegetables, greens and beans, seafood, and plant proteins. Participants in Bean Dietary Pattern 1 and 2 consumed greater amounts of total frut and whole fruit in comparison to the no bean dietary pattern, as indicated by the higher scores in these food categories. Adults in Bean Dietary Pattern 1, 2, and 3 consumed greater quanities of whole grain relative to the no bean group. Adults consuming Bean Dietary Pattern 1, 2, and 4 showed improved fatty acid ratios, which considered levels of polyunsaturated fatty acids (PUFAs), monounsaturated fatty acids (MUFAs) and saturated fatty acids (SFAs) when compared to the no bean group (standard for maximum score [PUFAs + MUFAs/SFAs] ≥ 2.5; standard for minimum score of zero [PUFAs + MUFAs/SFAs] ≤ 1.2).

**Table 3:**
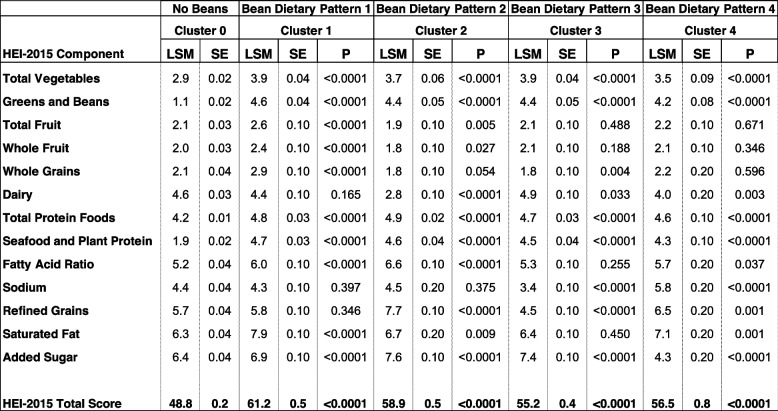
Adjusted mean (SE) healthy eating index-2015 total and component scores for all bean and no-bean clusters

Covariates include age, gender, ethnicity, poverty income ratio; NHANES 2001–2018, ≥ 19 Years-Old

*LSM* Least square mean, *SE* Standard error, *P p* value of difference as compared to cluster 0 (no beans)

When considering nutrients or food groups to moderate, added sugar scores (standard for maximum score ≤ 6.5% of energy; standard for minimum score of zero ≥ 26% of energy) were significantly better in 3 of the 4 bean patterns of consumption (Dietary Pattern 1, 2, and 3) relative to the no-bean group, while Bean Dietary Pattern 4 had a higher added sugar contribution, likely due to the large added sugar contribution from sweets and snacks (see Table 1). Saturated fat contribution from 3 of the 4 bean patterns of consumption was greater (standard for maximum score ≤ 8% of energy; standard for minimum score of zero ≥ 16% of energy) versus the no bean group, while Bean Dietary Pattern 3 was not statistically different from the no bean group. Refined grains (standard for maximum score ≤ 1.8 oz equivalents per 1,000 kcal; standard for minimum score of zero ≥ 4.3 oz equivalents per 1,000 kcal) provided significantly less energy in Bean Dietary Pattern 2 and 4, while Bean Dietary Pattern 3 contributed a greater amount of refined grains relative to the no bean group. Sodium scores (standard for maximum score ≤ 1.1 g per 1,000 kcal; standard for minimum score of zero ≥ 2.0 grams1,000 kcal) were poorer in Bean Dietary Pattern 3 and improved in Bean Dietary Pattern 4 compared to the no bean group.

### Fruit, vegetables, grain and fat intake within bean patterns of consumption

The mean intake of various FPED food groups within the dietary patterns of consumption identified are summarized in Table 4. Total fruit consumption was 30% higher in the greatest bean consumption pattern (Bean Dietary Pattern 1) relative to the no bean group (1.3 ± 0.06 vs. 1.0 ± 0.02 cup equivalents; *p* < 0.0001). Adults consuming Bean Dietary Pattern 2 had lower total fruit consumption versus the no bean group. Total vegetable consumption was 20% greater when comparing Bean Dietary Pattern 3 to the no beans group (1.8 ± 0.05 vs. 1.5 ± 0.01 cup equivalents; *p* < 0.0001). Refined grain consumption was only lower in Bean Dietary Pattern 3, while whole grain consumption was significantly higher in Bean Dietary Pattern 1 and 4, such that adults showed 57% increased whole grain consumption in both bean patterns of consumption in comparison to the no beans group (1.1 ± 0.06 vs. 0.7 ± 0.01 oz equivalents; *p* < 0.0001; and 1.1 ± 0.2 vs. 0.7 ± 0.01 oz equivalents; *p* = 0.019). Consumption of oils were significantly elevated in adults consuming Bean Dietary Patterns 2, 3 and 4 compared to the no beans group, however, these bean consumers also had significantly higher intake of monounsaturated and polyunsaturated fatty acids (see Table 2). Solid fats were significantly lower in Bean Dietary Pattern 1 (27.7 ± 0.8 vs. 35.3 ± 0.3 g; *p* < 0.0001) relative to the no beans pattern of consumption, while solid fats were significantly higher in Bean Dietary Pattern 3 and 4, likely due to the higher calories stemming from mixed dishes and sweets and snacks (see Table 1).

**Table 4:**
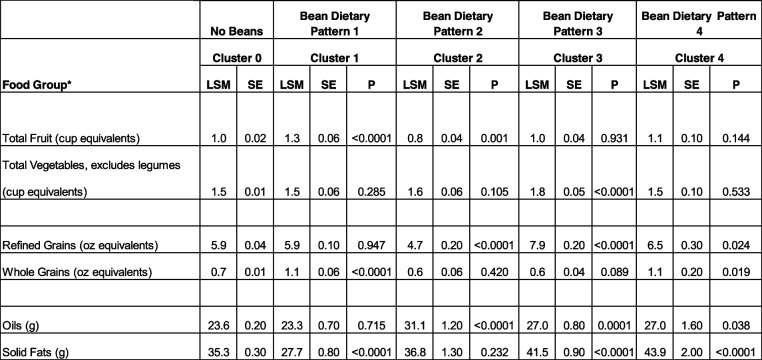
Adjusted mean (SE) fruit, vegetables, grains, and fat intake for all bean and no-bean clusters

Covariates include age, gender, ethnicity, poverty income ratio; NHANES 2001–2018, ≥ 19 Years-Old

*LSM* Least square mean, *SE* Standard error, *P p* value of difference as compared to cluster 0 (no beans)

### Weight-related health outcomes within bean patterns of consumption

Mean body mass index (BMI), body weight and waist circumferenes in the bean dietary patterns examined are summarized in Table 5. Compared to the No Beans pattern, adults consuming Bean Dietary Pattern 1 and Bean Dietary Pattern 2 demonstrated significantly lower BMI, reduced body weight, and smaller waist circumference. No associations were oberved in when examining Bean Dietary Pattern 3 and 4 relative to the No Beans dietary pattern, however, adults in Bean Dietary Pattern 4 approached significance for BMI (*p* = 0.058).

**Table 5:**
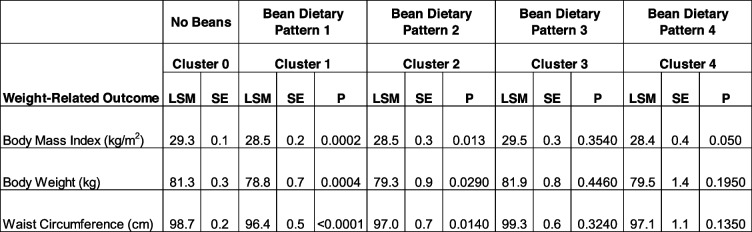
Adjusted mean weight-related health outcomes for all bean and no-bean clusters

Covariates include age, gender, ethnicity, poverty income ratio; NHANES 2001–2018, ≥ 19 Years-Old

*LSM* Least square mean, *SE* Standard error, *P p* value of difference as compared to cluster 0 (no beans)

## Discussion

Several bean dietary patterns of consumption in US adults were associated with favorable nutrient intakes. Bean consumers showed higher intakes of several shortfall nutrients (choline, alpha-linolenic acid, folate, iron, magnesium and vitamin E) relative to no bean consumption. Similarly, intake of dietary fiber, potassium and calcium, all nutrients of public health concern were significantly elevated in bean patterns relative to avoiding beans in dietary patterns. Two of the bean-rich dietary patterns were also significantly associated with improved weight-related measures, such that adults consuming Bean Dietary Pattern 1 and 2 had lower BMIs (-0.8 kg/m^2^ and kg/m^2^, respectively), reduced body weight (-2.5 kg and -2.0 kg, respectively) and smaller waist circumferences (-2.3 cm and -1.7 cm) in comparison to adults in the no-bean group. Dietary patterns that are rich in beans were associated with significantly higher diet quality scores, predominantly due to elevated scores from food groups encouraged by DGA, including total vegetables, greens and beans, seafood, and plant proteins. The current findings suggest that avoiding beans within dietary patterns may lead to nutrient and public health consequences in adults. Upcoming dietary guidance should consider the health benefits associated with the promotion of increased bean consumption in dietary patterns and develop strategies to encourage increased consumption in American adults.

The current analysis is aligned with a previous study using data from NHANES 1999–2002, where various types of canned and dry bean consumption were associated with improved nutrient intakes and favorable health outcomes [12]. Shared findings between the various groups of bean consumers showed that irrespective of the type of beans consumed, bean consumption was associated with greater dietary fiber and potassium intakes versus non-bean consumption. When considering baked beans, a very popular form of bean consumption in American dietary patterns, adult consumers had higher intakes of several shortfall nutrients, including dietary fiber, potassium, magnesium, and iron compared to bean non-consumers. Further, adult baked bean consumers had significantly lower systolic blood pressure versus non-consumers, concurrent to greater sodium intake. In the same analysis, adults consuming a variety of beans (i.e., pinto bean, kidney beans, etc.) also had higher intakes of dietary fiber, potassium, magnesium, iron, and total folate in comparison to non-consumers. Adults consuming a variety of beans showed lowered body weights, reduced waist circumferences and a 29% lower risk of having an elevated waist circumference compared to non-consumers. When consumption of baked beans and variety beans were combined in the analysis, adult consumers had a 23% lowered risk of increased waist circumference and a 22% reduced risk of having obesity relative on non-consumers [12].

The current work consistently showed that all bean-inclusive dietary patterns were associated with higher diet quality scores relative to non-bean consumption, as much as 25% higher. This finding is consistent with evidence presented in the 2020 DGAC. Specifically, higher diet quality scores have been documented among Asian Americans predominantly propelled by higher intakes of fruits, vegetables, greens and beans concurrent to reduced added sugar and saturated fat intake [1]. Diet quality has consistently been reported as a key component of health outcomes, quality of life and longevity. For instance, a recent review described strong substantiation stemming from prospective cohort trials where higher diet quality scores were related to a 14–29% reduced risk of cardiovascular disease and 0.5–2.2 years greater cardiovascular-free survival time [31]. Similarly, evidence from the Women’s Health Initiative Observational Cohort Study demonstrated 23% reduced cardiovascular disease risk and 30% lowered risk of heart failure in individuals with the greatest diet quality scores [32]. Data from nearly 39,000 men in the Health Professional’s Follow-Up Study cohort and 68,000 women from the Nurses’ Health Study cohort demonstrated significant multivariate risk reductions related to diet quality scores, such that improved diet quality (i.e., higher diet quality scores) was associated with 39% and 19% decreased risk of cardiovascular disease in men and women, respectively [33]. Similarly, another analysis using data from Health Professional’s Follow-Up Study cohort and the Nurses’ Health Study found participants in the top diet quality score quintile had an overall 19% lowered risk for major chronic disease. Further, the investigators showed the highest diet quality score quintile to be associated with 24% decreased risk of cardiovascular disease, 31% reduced risk for coronary heart disease, 20% lowered risk for stroke, 23% decreased risk for diabetes, and 6% reduced risk for cancer [33]. Likewise, the Mediterranean Diet Score, which encompasses high consumption of legumes amongst other components, found that higher scores were related to significant reductions in all-cause, coronary heart disease and cancer mortality rates in a Greek cohort of approximately 22,000 men and women. Indeed, a greater adherence to a Mediterranean diet was associated with 25%, 33% and 24% reduced risk of all-cause, coronary heart disease and cancer mortality, respectively [34]. The Global Burden of Disease Study [35] recently further emphasized the prominence of diet quality and associations to cardiovascular disease-related mortality outcomes. In particular, of thirteen dietary risk factors examined for cardiovascular disease mortality risk, only a high sodium consumption, a low intake of whole grains and reduced intake of legumes contributed significantly to age-adjusted mortality and disability-adjusted life years, leading the investigators to recommend reducing sodium, and increasing consumption of whole grains and legumes as the top global priority to improve diet quality and significantly reduce cardiovascular disease burden [35]. The relationship between diet quality and chronic disease outcomes is evident with the revisions and updating of the HEI-2015 scale via collaborations involving the USDA and the National Cancer Institute [18]. While the majority of the dietary components were unaltered, a major amendment involved the procedure by which legumes were assigned to the food components of the HEI scale. Specifically, in HEI-2015, which represents the most recent version of the diet quality scale, legumes are distributed to four of the thirteen components of which includes ‘Total Protein Foods’, seafood and Plant Proteins, ‘Total Vegetables’, and ‘Greens and Beans’ [18].

DGA 2020 advocates that Americans consume below 2300 mg/day of sodium as part of a healthy dietary pattern [4]. Nevertheless, approximately 9 in 10 Americans ≥ 2 years-old consume excessive levels of sodium daily [36], with average sodium intakes being greater than 3400 mg per day [37]. In the present analysis, while sodium levels were higher in three bean dietary patterns of consumption, relative to the no-bean group, potassium intake levels were significantly higher in all bean patterns of consumption examined. An important, and often missed principle, involves potassium intakes when assessing sodium intakes in the American population. When the US Congress asked the CDC to assess and review the Dietary Reference Intakes for sodium, the CDC decided to combine sodium and potassium into one assessment given the clear and established interrelationship of the two nutrients and the evident role sodium and potassium contribute to public health [38]. The American Heart Association has previously stated “foods with potassium can help control blood pressure by blunting the effects of sodium and the more potassium you eat, the more sodium you process out of the body. Potassium also helps relax blood vessels, which helps lower blood pressure” [39, 40]. It is also important to note that higher sodium contributions in Bean Dietary Pattern 2, 3 and 4 were likely contributed from the greater intake of sweets/snacks and mixed dishes (see Table 1). In addition, canned beans can contribute greater sodium, however, a substantial amount of sodium can be eliminated via draining the brine and rinsing the beans prior to consumption. Nonetheless, as it has been documented that Americans consume greater amounts of canned beans vs. dry beans [11], it is logical to assume that the present positive nutritional findings, as well as NHANES-based results from previously published data [12], are driven to a substantial extent from canned bean consumption, irrespective of sodium levels. Indeed, other researchers using NHANES datasets highlighted the nutritional benefits of including both dried beans and drained canned beans as a strategy to promote healthy dietary patterns [41]. Future research should consider bean patterns of consumption and various health outcomes, including blood pressure and cardiovascular outcomes that have been linked to sodium intake.

Limitations with NHANES datasets have been previously reported [42–44]. Nutrient intake and diet quality data are obtained from 24-h dietary recalls, which rely on study participant memory. While validated procedures are used to collect the data, recalled information may be inaccurate and biased from misreporting or memory challenges [45, 46]. In addition, the current evidence, being observational, cannot establish a causal link between the different bean dietary patterns examined and improvements in nutrient intakes and diet quality. However, a significant and robust advantage of the current work stems from the use of NHANES, which is a large continuous survey that examines a nationally representative sample of about 5,000 individuals yearly by highly-trained medical personnel [29]. Additionally, numerous covariates were used to adjust the data in an attempt to remove potential confounding scenarios. For the diet quality analysis, the simple algorithm and code available to the public was used in the analysis, and day-to-day variability may pose as a limitation, however, the large sample size of the current analysis helps mitigate this issue.

## Conclusions

The present analyses demonstrated favorable associations with bean consumption, shortfall nutrient intakes, weight outcomes, and diet quality. Adults consuming bean dietary patterns had higher intakes of several shortfall nutrients compared to no bean consumption, and greater intake of dietary fiber, potassium and calcium, all nutrients of public health concern. Two of the bean dietary patterns identified were further associated with significant improvements in weight-related measures, including BMI, body weight and waist circumference, relative to dietary patterns with no beans. All bean dietary patterns of consumption were associated with significantly higher diet quality scores, predominantly due to elevated scores from food groups encouraged by DGA, including total vegetables, greens and beans, seafood, and plant proteins. The avoidance of beans within dietary patterns may exacerbate current nutrient shortfalls and may even lead to nutrient and public health consequences in adults. Dietary strategies involving the promotion of increased canned and dry bean consumption within dietary patterns may prove to have numerous public health benefits for American adults.

## Data Availability

Publicly available US datasets were analyzed in the present study and can be found here https://wwwn.cdc.gov/nchs/nhanes. The NHANES datasets analyzed during the current study are available from the corresponding author on request.

## References

[1] Plant-based diets and their impact on health (2021). sustainability and the environment: a review of the evidence: WHO European Office for the Prevention and Control of Noncommunicable Diseases.

[2] Aguirre-Sánchez L, Teschner R, Lalchandani NK, El Maohub Y, Suggs S (2023). Climate change mitigation potential in dietary guidelines: A global review. Sustainable Prod Consump.

[3] Stagnari F, Maggio A, Galieni A, Pisante M (2017). Multiple benefits of legumes for agriculture sustainability: An overview. Chem Biol Tech Agric.

[4] US Department of Agriculture (2020). U.S. Department of Health and Human Services. Dietary Guidelines for Americans, 2020–2025.

[5] US Department of Health and Human Services (2015). U.S. Department of Agriculture. 2015–2020 Dietary Guidelines for Americans.

[6] United States Department of Agriculture. Agricultural Research Service USDA Food Composition Databases, USDA National Nutrient Database for Standard Reference (Release 28, Released September 2015, Revised April 2019). Available online: https://fdc.nal.usda.gov. Accessed 7 Apr 2023.

[7] Comerford KB, Papanikolaou Y, Jones JM, Rodriguez J, Slavin J, Angadi S, Drewnowski A (2021). Toward an evidence-based definition of and classification of carbohydrate food quality: An expert panel report. Nutrients.

[8] Drewnowski A, Maillot M, Papanikolaou Y, Jones JM, Rodriguez J, Slavin J, Angadi S, Comerford KB (2022). A new carbohydrate food quality scoring system to reflect dietary guidelines: An expert panel report. Nutrients.

[9] Comerford KB, Drewnowski A, Papanikolaou Y, Jones JM, Slavin J, Angadi S, Rodriguez J (2023). Application of a new carbohydrate food quality scoring system: An expert panel report. Nutrients.

[10] US Department of Agriculture, Agricultural Research Service (2018). Food patterns equivalents intakes from food: mean amounts consumed per individual, by gender and age, what we eat in America, NHANES 2015–2016.

[11] Proprietary data calculated by Bush’s Best and based in part on data reported by NIQ through its Syndicated database for the Bean category (branded only) for the Latest 52 weeks ending 4/29/23, for Total US xAOC (sales in the Food, Drug, Mass, Club, Dollar, and Military channels), according to the NIQ standard product hierarchy. Copyright © 2023, Nielsen Consumer LLC.

[12] Papanikolaou Y, Fulgoni VLF (2008). Bean consumption is associated with greater nutrient intake, reduced systolic blood pressure, lower body weight, and a smaller waist circumference in adults: Results from the National Health and Nutrition Examination Survey 1999–2002. J Am Coll Nutr.

[13] NchanjiBogweh E, Ageyo Collins O (2021). Do common beans (Phaseolus vulgaris L.) promote good health in humans? A systematic review and meta-analysis of clinical and randomized controlled trials. Nutrients.

[14] Bazzano LA, He J, Ogden LG, Loria C, Vupputuri S, Myers L, Whelton PK (2001). Legume consumption and risk of coronary heart disease in US men and women: NHANES I epidemiological follow-up study. Arch Intern Med.

[15] Cryne CN, Veenstra JM, Deschambault BR, Benali M, Marcotte M, Boye JI, Tosh SM, Farnworth ER, Wright AJ, Duncan AM (2012). Spray-dried pulse consumption does not affect cardiovascular disease risk or glycemic control in healthy males. Food Res Int.

[16] Drewnowski A (2005). Concept of a nutritious food: Towards a nutrient density score. Am J Clin Nutr.

[17] Drewnowski A (2010). The nutrient rich foods index helps to identify healthy, affordable foods. Am J Clin Nutr.

[18] National Cancer Institute, Division of Cancer Control & Population Sciences. Epidemiology and Genomics research program. Developing the healthy eating index. Available online: https://epi.grants.cancer.gov/hei/developing.html. Accessed 10 Apr 2023.

[19] López-González L, Becerra-Tomás N, Babio N, Ángel Martínez-González M, Díaz-López A, Corella D (2021). Variety in fruits and vegetables, diet quality and lifestyle in an older adult Mediterranean population. Clin Nutr.

[20] Keim NL, Forester SM, Lyly M, Aaron GJ, Townsend MS (2014). Vegetable variety is a key to improved diet quality in low-income women in California. J Acad Nutr Diet.

[21] Garcia-Bailo B, Jain N, Keeler C, Smith J. Legume consumption, diet quality and body weight: Results from NHANES 2009–2012 and the food patterns equivalent database 2009–2012. FASEB J. 2018. 10.1096/fasebj.31.1_supplement.648.15

[22] Zargarzadeh N, Mohammad Mousavi S, Santos HO, Aune D, Hasani-Ranjbar S, Bagher Larijani B, Esmaillzadeh A (2023). Legume consumption and risk of all-cause and cause-specific mortality: A systematic review and dose-response meta-analysis of prospective studies. Adv Nutr.

[23] Marventano S, Izquierdo Pulido M, Sánchez-González C, Godos J, Speciani A, Galvano F, Grosso G (2017). Legume consumption and CVD risk: A systematic review and meta-analysis. Public Health Nutr.

[24] US Centers for Disease Control and Prevention; National Center for Health Statistics; National Health and Nutrition Examination Survey. NHANES—National Health and Nutrition Examination Survey Homepage. Available online: https://www.cdc.gov/nchs/nhanes/index.htm. Accessed 17 Sept 2021.

[25] United States Department of Agriculture; Agricultural Research Service. Food Service Research Group. What We Eat in America, National Health and Nutrition Examination Survey Overview, 2001–2018: Data Collection. Beltsville: United States Department of Agriculture, Agricultural Research Service; 2021. Available online: https://www.ars.usda.gov/northeast-area/beltsville-md-bhnrc/beltsville-human-nutrition-research-center/food-surveys-research-group/docs/wweianhanes-overview/. Accessed 19 Sept 2021.

[26] Centers for Disease Control and Prevention. National Center for Health Statistics. National Health and Nutrition Examination Survey. Available online: https://www.cdc.gov/nchs/nhanes/index.htm. Accessed 17 Apr 2023.

[27] US Department of Agriculture; Agricultural Research Service; Food Surveys Research Group. Food and Nutrient Database for Dietary Studies. Available online: https://www.ars.usda.gov/northeast-area/beltsville-md-bhnrc/beltsville-human-nutrition-research-center/food-surveys-research-group/docs/fndds-download-databases/. Accessed 17 Sept 2021.

[28] Centers for Disease Control and Prevention; National center for health statistics. National health and nutrition examination survey. Analytic and reporting guidelines. Available online: https://wwwn.cdc.gov/nchs/nhanes/analyticguidelines.aspx. Accessed 17 Sept 2021.

[29] Moshfegh AJ, Rhodes DG, Baer DJ, Murayi T, Clemens JC, Rumpler WV, Paul DR, Sebastian RS, Kuczynski KC, Ingwersen LA (2008). The USDA automated multiple-pass method reduces bias in the collection of energy intakes. Am J Clin Nutr.

[30] USDA Ag Data Commons. US Department of Agriculture. Food and Nutrient Database for Dietary Studies (FNDDS). Available online: https://data.nal.usda.gov/dataset/food-and-nutrient-database-dietary-studies-fndds. Accessed 12 May 2023.

[31] Petersen KS, Kris-Etherton PM (2021). Diet quality assessment and the relationship between diet quality and cardiovascular disease risk. Nutrients.

[32] Belin RJ, Greenland P, Allison M, Martin L, Shikany JM, Larson J, Tinker L, Howard BV, Lloyd-Jones D, Van Horn L (2011). Diet quality and the risk of cardiovascular disease: The Women’s Health Initiative (WHI). Am J Clin Nutr.

[33] McCullough ML, Feskanich D, Stampfer MJ, Giovannucci EL, Rimm EB, Hu FB, Spiegelman D, Hunter DJ, Colditz GA, Willett WC (2002). Diet quality and major chronic disease risk in men and women: Moving toward improved dietary guidance. Am J Clin Nutr.

[34] Trichopoulou A, Costacou T, Bamia C, Trichopoulos D (2003). Adherence to a Mediterranean diet and survival in a Greek population. N Engl J Med.

[35] Zhang B, Pu L, Zhao T, Wang L, Shu C, Xu S, Sun J, Zhang R, Han L (2023). Global burden of cardiovascular disease from 1990–2019 attributable to dietary factors. J Nutr.

[36] Jackson SL, Coleman King SM, Zhao L, Cogswell ME (2016). Prevalence of sodium intake in the United States. MMWR.

[37] Cogswell ME, Loria CM, Terry AL, Zhao L, Wang CY, Chen TC (2018). Estimated 24-hour urinary sodium and potassium excretion in US adults. JAMA.

[38] National Academies of Sciences, Engineering, and Medicine 2019. Dietary reference intakes for sodium and potassium. Washington, DC: The National Academies Press. Available online: 10.17226/25353, http://nap.edu/25353. Accessed 7 May 2023. 30844154

[39] American Heart Association. How potassium can help control high blood pressure. Available online: https://www.heart.org/en/health-topics/high-blood-pressure/changes-you-can-make-to-manage-high-blood-pressure/how-potassium-can-help-control-high-blood-pressure. Accessed 10 May 2023.

[40] American Heart Association. A Primer on potassium. Available online: https://www.heart.org/en/healthy-living/healthy-eating/eat-smart/sodium/potassium. Accessed 12 May 2023.

[41] Zanovec M, O’Neil CE, Nicklas TA (2011). Comparison of nutrient density and nutrient-to-cost between cooked and canned beans. Food Nutr Sci.

[42] Ahluwalia N, Dwyer J, Terry A, Moshfegh A, Johnson C (2016). Update on NHANES dietary data: focus on collection, release, analytical considerations, and uses to inform public policy. Adv Nutr Int J.

[43] Zipf G, Chiappa M, Porter K, Ostchega Y, Lewis B, Dostal J (2013). The national health and nutrition examination survey: plans and operations. Vital Health Stat.

[44] Grandjean AC (2012). Dietary intake data collection: Challenges and limitations. Nutr Rev.

[45] Ferrari P, Slimani N, Ciampi A, Trichopoulou A, Naska A, Lauria C, Veglia F, Bueno-de-Mesquita HB, Ocké MC, Brustad M (2002). Evaluation of under- and overreporting of energy intake in the 24-hour diet recalls in the European Prospective Investigation into Cancer and Nutrition (EPIC). Public Health Nutr.

[46] Dwyer J, Picciano MF, Raiten DJ, Members of the Steering Committee (2003). Collection of food and dietary supplement intake data: What we eat in America—NHANES. J Nutr.

